# Working with families of adults affected by eating disorders: uptake, key themes, and participant experiences of family involvement in outpatient treatment-as-usual

**DOI:** 10.1186/s40337-022-00611-z

**Published:** 2022-06-29

**Authors:** Carmel Fleming, Jacqueline Byrne, Karen Healy, Robyne Le Brocque

**Affiliations:** 1 School of Nursing, Midwifery, and Social Work, The University of Queensland, Brisbane, Qld 4072 Australia; 2 Queensland Eating Disorder Service, Queensland Health Metro North Hospital and Health Service, Brisbane, Australia; 3 School of Nursing and Midwifery, Griffith University, Brisbane, Qld 4122 Australia

**Keywords:** Eating disorders, Adults, Families, Carers, Family involvement, Single session family consultation

## Abstract

**Background:**

Eating disorders are associated with significant personal and family costs. Clinical guidelines recommend family members be involved and supported during care, but little has been reported regarding the preferences of adults around carer involvement in treatment. The necessary intensity of family work with adults is also unknown. A trial of a standardised brief family involvement method was conducted in an adult eating disorder service offering treatment-as-usual. Uptake and feasibility of implementing the approach as part of standard outpatient care and the preliminary impact on issues identified by adult patients and carers were evaluated.

**Methods:**

Eligible referrals at an adult eating disorders outpatient clinic were offered as needed family consultation to address presenting interpersonal problems identified by patients and their family members, and outcomes were evaluated 4 weeks later. Pre and post intervention surveys identified participant self-reported change in (i) problem frequency, (ii) distress and disruption caused, and (iii) confidence regarding presenting problems. Open text responses provided an overview of patient and carer goals for family involvement and revealed how the novel method impacted these areas as well as overall experience of, and feedback regarding, the brief family intervention.

**Results:**

Twenty-four female participants aged 18–53, and 22 carers participated in 31 consultations. Common concerns raised were eating disorder related interpersonal and communication issues. The focused sessions, offered on a one-at-a-time basis, showed preliminary effectiveness for reducing both patients and carer concerns. For example, adult patients reported that life interference from interpersonal problems was lower and confidence to deal with them was higher following family consultation. Carers also reported that frequency, level of worry, and life interference around presenting problems were lower after the structured family intervention.

**Conclusions:**

Brief family consultation, with a single focus on issues identified by family members and adult patients, was a safe and feasible procedure with adults affected by eating disorders. Effective at meeting the needs of participants, the framework investigated in the current study may also be a useful direction for adult services to consider when looking to support families and meet recommendations for their routine involvement in the outpatient care.

*Trial registration*: Australian Clinical Trials Register number: ACTRN12621000047897 (www.anzctr.org.au).

## Plain English Summary

Eating disorders are serious problems that can have negative consequences for both the person affected and their family members. Research shows that family involvement can support treatment, but little is known about whether adults with eating disorders want their families involved or how much. This study asked if adding a brief family intervention to normal treatment would be safe, viable and effective. The uptake of, and impact from, a one-off family consultation was measured in 24 adult patients and 22 carers who identified the problems they were facing as a result of the eating disorder, how often these were happening, how worried they felt about them, and how much the issues were interfering in their life before and after the family session. Both the patients and carers reported change in these areas. Issues regarding the eating disorder and communication were the most common concerns of adult patients and carers. Feedback about the sessions was positive and one session was enough to address the immediate concerns for many families. Single session family consultation may be a promising option for services working with the families of adults affected by eating disorders to consider in the future.

## Introduction

Eating disorders (EDs) in adults are complex and difficult to treat mental health conditions. Issues of patient motivation, preferences, and commitment to treatment are known concerns when working with adults affected by EDs that can make achieving positive therapeutic change difficult [1–4]. In the treatment of child and adolescent EDs family-based approaches are regarded as optimal for attaining best outcomes and have been comprehensively described in the literature [5–7]. In contrast, the descriptions of family inclusive treatment methods, and the content and outcomes of these, for adults is limited [8]. It is only recently that joint sessions between the family and adult patient have been routinely reported as part of good-quality care-as-usual, although the detail of what such joint sessions include or how acceptable they are to participants is rarely described (for example, [9]). This is despite the fact that patients consistently identify direct support and encouragement from others as central to recovery and perhaps even a ‘driving force’ in the process [10, 11].

Given the interpersonal difficulties that people with EDs can experience, it is surprising that it is only relatively recently that trials into family interventions for adult ED populations have started to be reported and the value of relational approaches with adults considered [12–15]. Integrated family treatment approaches that have been evaluated for adults include: a cognitive-interpersonal treatment for anorexia nervosa,adult applications of family-based therapy as used with adolescents; couple-based interventions; and multifamily group therapy approaches for adults [16–26].

This is perhaps understandable given that adult treatment guidelines have only recently included recognition of the fact that family members affected by EDs should be identified, consulted, and supported [27, 28]. Such guidelines specify several relational tasks for clinicians as part of best-practice care. For example, the NICE guideline states ED practitioners need to: ‘Find out what… family members or carers (as appropriate) know about eating disorders and address any misconceptions’,‘Offer people with an eating disorder and their family members or carers (as appropriate) education and information’; ‘Assess the impact of the home…and social environment…on each person’s eating disorder’; ‘Encourage family members, carers, …of children and young people to support them during their treatment’; and ‘Be aware that the family members or carers of a person with an eating disorder may experience severe distress. Offer family members or carers assessments of their own needs as treatment progresses’. There is however little detail as to exactly how clinicians might approach these tasks or of indicated methods to achieve such tasks. As noted, family-based treatment is indicated for adolescent cases but there is no consensus as to what constitutes effective family inclusion of family members in the treatment for adults with EDs.

Other areas of serious mental illness have a longer history of developing and testing family inclusive treatments for adults that facilitate the structured involvement of carers, patient choice, and collaborative decision-making [29]. Brief relational approaches have also been found to be effective for supporting the families of adults affected by other mental illnesses [30]. Many take a solution-focused, strengths-based approach, which have been found to assist with negative expectations regarding family involvement that can exist [31, 32]. In early psychosis, depression, and bipolar disorders family work of varying intensity has been found to improve clinical outcomes and is strongly recommended in treatment guidelines [33–35]. Even family work via one-off sessions, delivered on an as-needed basis, have been demonstrated to be useful in other serious mental health issues [36–38].

Of the small number of low intensity or brief family interventions to have been developed in EDs, none involve adult patients (see [39–41]), something that may be particularly important given the need to support self-efficacy in this population [42, 43].

One method used in the broader mental health field, single session family consultation (SSFC), has been described as an option that combines the efficiency of single session treatment with the efficacy of family therapy [44]. SSFC is a brief, structured, relational intervention that is typically limited to between one and three sessions that aim to support carers in their role to assist the affected individual, provide direct assistance regarding current issues, and improve family service engagement [45]. The SSFC framework has been manualized and tested in a variety of areas [32, 45–48], and found to be effective for working with families affected by a range of mental health conditions whilst also incorporating the needs and participation of the adult patient [37, 49–52].

The aim of the current study was to test if a similar low-intensity family intervention could be safely and feasibly added to outpatient care of adult EDs. Given this approach has proven safe and effective for working with adult families affected by a range of equivalent serious mental illnesses, it was expected that the brief relational method would also be safe sufficient for families affected by EDs and would be effective at reducing patient- and carer-identified relational problems. This paper reports the results of a proof-of-concept trial of an ED specific adaptation of a brief family intervention offered alongside adult outpatient treatment-as-usual (TAU) delivered in a routine clinical setting.

## Methods

### Design

This study used a one-group pretest–posttest design to examine the feasibility of the brief family method in addition to standard care. All patients receiving outpatient treatment could opt to have family involvement, which was available at any point throughout their treatment program. Participant ratings for frequency of, impact from, and confidence regarding self-reported interpersonal issues were taken via a self-report survey, at baseline (pretest), and again up to one month after the intervention (posttest). All participants were recruited from a community ED outpatient clinic in Brisbane, Australia between September 2018 and October 2019. For further information about services provided at the study site see www.health.qld.gov.au/clinical-practice/referrals/statewide-specialist-services/queensland-eating-disorder-service-queds.

Based on the research question and pragmatic approach to enquiry, the study aimed to change as little as possible about how the family intervention had been typically provided with other mental health conditions. Eligibility criteria for inclusion in the study were kept minimal and were framed to ensure that typical treatment participants and their families were included. There was no allocation of participants to the trial condition, participants could self-select to have a family involvement or not. A transdiagnostic sample of outpatients were therefore accepted and existing clinicians were used to deliver the intervention. The study was granted independent ethics approval by the University of Queensland and the Human Research Ethics Committees of the Royal Brisbane and Women’s Hospital (2018001740 HREC/18/QRBW/365).

### Participants

#### Study population and context

The study was conducted at the Queensland Eating Disorder Service (QuEDS), a specialist state-wide publicly funded service that provides community EDs treatment for individuals aged over 16 years. At the time of the study, QuEDS provided evidenced based individual outpatient therapy via either Cognitive Behavioural Therapy Enhanced (CBT-E) or Specialist Supportive Clinical Management (SSCM) [53, 54]. QuEDS also offered a specialist ED Day Program (4 days per week, over an 8-week period) that included supported meal therapy, dietetic advice, insight-oriented and recovery-focused therapeutic groups, as well as psychiatric care as required [55]. In addition to the family consultation session all families in contact with the service were offered general treatment information and a link to community support services.

#### Patients and carers

The sample was recruited from a consecutive series of referrals of patients with a transdiagnostic range of EDs to the QuEDS outpatient clinic over a 12-month period. Diagnosis, converted into DSM-IV category, was established from clinical interview with a consultant psychiatrist, and ratified via patient eating disorders examination questionnaire responses (EDE-Q, [56]). Patients assessed as at medical risk needing higher level of care, or who were ineligible to participate in the research due to lack of capacity to offer informed consent (subject to involuntary treatment orders), were excluded. Patients unable to identify any current support person or family contact were also excluded. The remaining voluntary, adult patients that began outpatient treatment-as-usual were approached to be recruited by a research assistant not attached to the clinical team. After informed consent, all patients completed a range of standard pre-treatment clinical questionnaires. Following discussion with their individual therapist, patients opting to have family involvement and their carers completed study questionnaires prior to, and after, the family consultation.

#### Clinicians

Over the study period, 14 clinicians were involved in delivering the family intervention at the research site including four clinical psychologists, three mental health social workers, two mental health occupational therapists, two clinical psychology trainees, one clinical nurse consultant, and one psychiatry registrar. A mental health social worker, trained in SSFC and the first author, cofacilitated all the sessions for consistency. A half day orientation to the framework was provided to clinicians who were all experienced in delivering the treatment-as-usual options but had no prior experience in the use of the brief family consultation method. Clinical supervision of the sessions was provided by a senior clinician from the sponsoring hospital and health service who had also been trained in SSFC for other mental health conditions and was not part of the treating team.

### Procedure

#### Treatment-as-usual

TAU at the research site consisted of (i) a 20–40-week course of outpatient treatment, either CBT-E, SSCM, or (ii) an eight-week day program as described above.

#### Family intervention

Originally developed for mental health services and refined by American and Australian family practitioners, the intervention used was based on a brief family engagement process where 1–3 meetings are conducted between the primary patient and family members [32, 45–47, 57–59]. The focus of the consultation is negotiated with the patient prior to the session and further refined with the whole family during the session. Other steps in conducting the consultations include formulating a shared view of current issues for the family, clarifying the nature of family involvement in the individual’s treatment, assisting carers to identify and respond to their own needs.

The planned meeting was held in person or via phone or video conferencing if necessary. The session included the individual patient and any key support people they chose to involve, as well as the patient’s individual treating clinician and the family worker. Two further sessions could be scheduled during the treatment period if requested. If the consultation identified patient or family concerns that were beyond the remit of the session or the treatment team, referral to specialised family services was offered. One month following, participants were contacted in person, by phone, or email regarding any additional requirements or need for further intervention.

### Measures

Feasibility, defined as the degree that a clinical innovation is used successfully or can be carried out within a given setting, was indicated by rates of recruitment, uptake, and participation in the method [60, 61]. The impact of the intervention was established by comparing aggregated participant scores on identified issues from baseline to follow-up. Directly prior to, and up to 1-month following the joint family meeting, patients and carers completed a written questionnaire, the Problem Evaluation Summary (PES), that was also used as an aid to set the agenda for sessions and then explore the degree to which participant needs had been met [45]. Ratings of how frequently identified issues were occurring, self-reported distress around relational issues (how worried participants were about the problems), levels of disruption caused (how much the problems interfered with their lives), and confidence to deal with the identified issues were indicated via a 10-point Likert scale. Participants were also asked, ‘If this family session was successful, what would you and your family be doing differently?” to further discern session goals. In addition to repeating the frequency, distress, disturbance and confidence measures, the post-session questionnaire asked patients and carers to identify the main things that had and had not changed since the session. Further open-ended survey questions asked if additional support was required or if participants had other comments they wished to make.

### Analysis

The Statistical Package for the Social Sciences ([62], version 23 for Windows) was used to provide descriptive statistics and analyse the participant ratings. Repeated measures t‐tests were used to compare pre-post scores and a measure of effect size (Cohen’s d) was calculated to estimate the degree of change in the patient and the carer group for participant-rated confidence, frequency, and impact of the major presenting problem identified by the PES. This was appropriate for a pre-post open trial as the analysis units had similar standard deviations and were of the same size. Sensitivity to change is important in small or diverse groups [63].

As the intervention had not been previously tested with ED patients, there were no prior data available for sample size calculations and the sample was determined by the number of referrals to the outpatient clinic who elected to participate. At the time of the study design, only one previous study on the effectiveness of using a single‐session family approach with carers of children and adolescents in an Australian mental health setting was available [32]. In the absence of a more comparable sample, the results of this research were used to benchmark a possible magnitude of effect and an informal sample size calculation, establishing that between 16 and 26 participants would be necessary to achieve power to detect a similar magnitude of change using the same intervention outcome survey in the current study. Similar sample sizes have been used in pilot studies and case series evaluations of other family interventions used with adult EDs cohorts [17, 18, 24, 25].

Given the lack of prior research on the application of brief family consultation with adults with EDs, inductive category development was used to code the open text participant responses [64]. Replies to each open-ended question were read through, key words denoting central concepts highlighted, and notes made regarding commonalities. Problem areas presented in the family sessions were analysed to assess the degree of convergence, common or discordant problem areas identified for patient and carer groups, and quantitative scores as well as open text responses synthesized to assess the acceptability and initial effectiveness of the brief family intervention for addressing participant issues [65, 66].

## Results

### Research participation, participant flow, and attrition

**Fig. 1:**
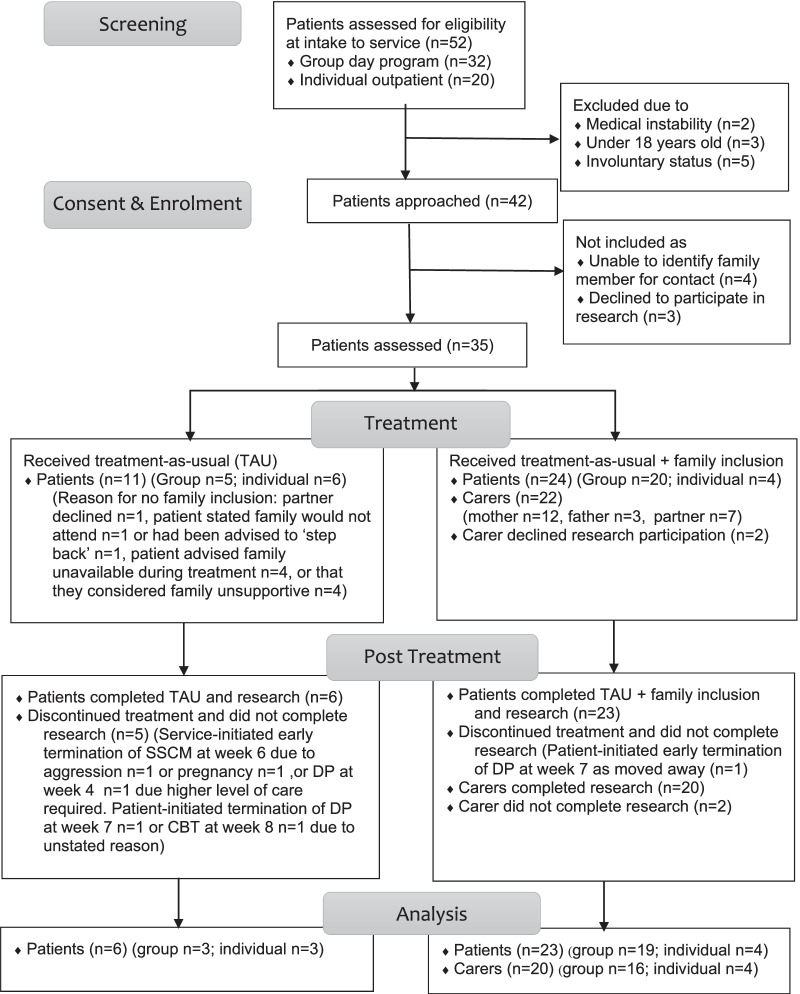
CONSORT diagram, describing the flow of participants through the study

Over the 12-month recruitment period, 52 consecutive referrals were received to the outpatient service (see Fig. 1).

Ten referrals were ineligible for recruitment to the research project. Of the 42 patients eligible for research participation, three declined citing time constraints, confidentiality concerns, or being not interested in completing paperwork. Four patients were unable to identify a supportive carer for the service to contact. The remaining 35 patients consented and were enrolled in the research. Eleven of these patients chose not to include any carers in their treatment. Carers of the remaining 24 patients were then approached to participate in the research. Twenty-two carers consented to the research protocol and two declined. Baseline clinical characteristics and demographic data are presented in Table 1.

**Table 1:** Baseline demographic and clinical characteristics of participants

Demographic and clinical characteristics	Patients Treatment-as-usual only				Patients TAU + family involvement				Carers			
	*n*	M	SD	Range	*n*	M	SD	Range	*n*	M	SD	Range
Age	11	34.64	14.67	19–56	24	27.63	8.52	18–53	22	51.32	15.51	22–71
*Gender*												
Female	10				24				13 (59%)			
Male	0				0				9 (41%)			
*Relationship*												
Mother									12 (55%)			
Father									3 (14%)			
Partner									7 (32%)			
Currently living together									15 (68%)			
*Eating disorder diagnosis*												
Anorexia Nervosa	5				13							
Bulimia Nervosa	3				4							
Other specified feeding and ED	3				7							
Body mass index		20.41	2.19	17.4–23.7		20.58	3.22	17–26.7				
Eating disorder global (EDEQ)		4.26	0.93	2.45–5.82		3.88	1.30	0.9–5.54				
Restraint		3.55	1.74	0.8–6.0		3.42	1.61	0–5.6				
Eating concern		4.24	1.61	1.4–6		3.61	1.39	0.8–5.6				
Shape concern		4.83	0.96	3.5–6		4.32	1.39	0.75–5.88				
Weight concern		4.0	1.22	1.8–5.6		3.97	1.59	0.2–5.8				
Clinical impairment (CIA)		31.27	10.89	11–48		34.46	8.03	14–48				

A total of 31 family sessions were conducted and 61 pre-session questionnaires, corresponding to a response rate of almost 100% for this measure (one each from the patient and the primary carer for the 31 sessions) were returned. The pre-session form was used for research purposes as well as to establish the agenda for the family consultation. Participants that had more than one family session provided a pre-session form each time and all of these were included in the analysis. As participants were only asked to complete the post-session form once, the 39 post-session forms returned represents a response rate of 85% (19/24 patients and 20/22 carers).

### Feasibility and acceptability of the intervention

Eighty-three per cent (35/42) of eligible adults in outpatient treatment consented to research participation and were provided with carer orientation and family psychoeducation materials. Thirty-one per cent (11/35) opted for treatment-as-usual only with no further family involvement over the course of their outpatient care. Four of these patients stated no family were currently available to attend, four expressed that the relationship with their carer was currently unsupportive, one patient indicated she did not want family involved as the ED was a very long-standing problem that had impacted family dynamics, one patient said her family had been advised by another treatment team to ‘step back’, and one patient reported her partner declined involvement after a long history of contact with other treatment providers.

Sixty-nine per cent (24/35) of adult patients chose to involve their family in their treatment-as-usual via participation in at least one single session family consultation. There was no attrition from the intervention, with all patients attending the scheduled meeting as planned. A distress protocol, established prior to each session between facilitators should any participant become unable to continue with the session, was not instigated during any consultation. Nor were any family consultations terminated prematurely.

Over the study period, 31 sessions were conducted, with 19 families having a single session, three families participating in two meetings, and two families having three consultations. Sessions were attended by different combinations of support people in three of these five cases. For example, one patient first involved her partner, and then her parents, and finally had another session with her partner and parents together. A single session was therefore sufficient for the majority (19/24, 79%) of the those who took up the family intervention.

### Identified priority problems

The most frequently identified problems by patients were: eating disorder-specific assistance (n = 19 mentions), communication issues (n = 16), emotional impact (n = 14), the need for support, understanding and validation (n = 11), effects on relationships (n = 7), future concerns and continuing progress (n = 7), and managing expectations (n = 2) with most patients mentioning multiple problems across their replies.

Carers identified a very similar same range of issues including eating disorder-specific concerns (n = 17 mentions), communication issues (n = 11), future concerns and continuing progress (n = 11), role in providing support and understanding (n = 9), emotional impact (n = 7), and effects on relationships including with other family members (n = 3). See Table 2 for exemplars.

**Table 2:** Major presenting problems identified by participants

Theme	Patient examples	Carer examples
Eating disorder assistance	Support around mealtimes, especially during the holidays. Balance between enough support and not ‘policing’	Good eating habits and a healthy weight which will help with her state of mind. Organise the eating disorder plan so {she} can keep getting help
Communication issues	Communicating more in times of a lot of stress, leads to problems and having arguments	Finding out what’s going on and what I need to know without sounding like an interrogator
Emotional impact	The impact my mental health has had on my family. I feel very guilty, I think that’s why I like to stay thinner or a certain size because I don’t want to embarrass them by being fat	Knowing how to not get angry at {partners} ED behaviour
Providing support	Getting support without feeling I’m being told what to do, with my experiences being validated	Learn how to be a better support for my partner from my partner
Relationship effects	Dieting in the past perhaps impacting on potential for healthiest relationship – i.e., shared relationship around food (would like it to be positive)	Helping in a way that keeps our relationship healthy
Future progress	Learning how to communicate my needs and emotions during recovery and moving forward. Speaking up on the bad days so I can prevent relapse	Once the program finishes, what resources are available to continue with progress made. How can I identify and help with any potential relapse?
Expectations	Not being able to carry out good behaviours at home	
Other family members		General moodiness when visiting. Affects the other children, especially the youngest

Many replies also included multiple issues reflecting the interconnected nature of the presenting problems. For example, Patient 5 indicated she was “Wanting to cease eating disorder behaviours. Deal with emotions in a healthy way and have family understand why I do what I do”, and another stated, “I want my mum to be less stressed. The comments she makes, e.g., (‘this is terrible,’ ‘why can’t you just get better’) makes me feel guilty and want to restrict” (Patient 3).

To establish a solution focus prior to the session, participants were asked, ‘If this family support session was successful, what would you and your family be doing differently?’. A total of 28 patient and 26 carer replies (from 31 sessions) were received in response to this question. Both patients and carers commonly identified that, if the family intervention was effective, their knowledge, understanding and support of, as well as the emotional and relational response to the ED problem, would be different. Most participants identified multiple goals. For example, one patient stated she want to, “Understand each other in this context better. Have constructive conversations about struggles and support when needed. Feel comfortable to communicate feelings whenever” (Patient 2). A carer wrote she would like, “Relaxed social times. Not walking on eggshells, less stress. Speaking without checking potential misunderstandings, more relaxed” (Mother, Carer 3).

Improvement to communication was the most common change that participants wanted to achieve from family consultation. Eighteen of the 28 patients noted they would like to achieve either less conflictual or more frequent and supportive communication. Eleven of the 26 carers expressed similar wishes. Making up more than half the items coded (29/48), this theme was subsequently divided into ‘avoidance’ or ‘approach’ goals [67, 68]. For example, one patient (11) described an approach goal as “Parents (and myself) would know how to work together better. Practical ideas on how parents can help me. I would know how my parents feel about my issues”. An avoidance goal identified by the partner of a patient (Carer 21) was “Spending less time and energy worrying about meals”. See Table 3 for more information on the communication goals identified by participants.

**Table 3:** Communication related goals identified by participants

Patient approach goals for communication	Carer approach goals for communication
Appropriate conversations More conversation around intimacy Talking more opening and honestly abut both of our feelings I would be communicating more effectively I would check in with {partner}. Start things off (talking about it) Have constructive conversations about struggles and support when needed Feel comfortable to communicate feelings whenever Hopefully our communication would be better Able to communicate difficult emotions, not only positive. Reach out more easily Being able to communicate better Be able to express our feelings without the fear of upsetting someone I am more open with {partner} and we can have discussions about my sprogress more openly	Talking more Talking more open about everything Communicating better in the moment Better communication Easier to talk about issues because they are open—we are on the same page Open communication or knowing when {daughter} is starting to feel uncomfortable in a situation More communication regarding the eating disorders Would talk more. {Daughter} would know this is something that I want to help with. Not a burden Communicating better etc

Patient avoidance goals for communication	Carer avoidance goals for communication
Not making comments on others food etc There wouldn’t be so many arguments My mother would not say triggering things Understanding what they can help with and that some things that are said can be more harmful than good Language at home would be different. Less arguments Would like for it to be a bit easier to communicate and not withdraw as much	Speaking without checking potential misunderstandings Less theft and lies

Emotional support was another commonly mentioned change goal identified by patients (16 mentions), although this was often noted in relation to the communication of feelings. For example, one patient stated her goal with her partner was, “Talking more opening and honestly about both of our feelings. We would both provide each other emotional support and our house would be a 100% safe space” (Patient 12).

Achieving clarity about the role carers could take and increasing the effectiveness of their efforts in relation to the ED was the next most frequently mentioned item for change. Noted 13 times, carers noted for example the need to learn, “Balancing being tough and giving good support” (Father, Carer 7) and “…implement new and effective techniques for supporting {partner} in a constructive way” (Partner, Carer 6).

More supportive assistance and improved knowledge and understanding about the ED, combined with a desire for improvement and developing a life beyond the ED were also endorsed by several patients and carers. For instance, one patient said, “Hopefully they [parents] will be able to give me the ability to choose my recover process and do it independently BUT also having them involved and getting support” (Patient 15). A carer hoped that she would “Have more confidence, and feel more relaxed about being away from {daughter} to travel etc. Most importantly, {daughter} would be able to enjoy life more” (Mother, Carer 13).

### Frequency of participant problems and levels of distress, disruption, and confidence

Means and standard deviations for the measures prior to the family consultation and at one-month follow-up are presented in Table 4, revealing the preliminary impact of participating in the family consultation for patients and carers. No differences were found between the families that had one session and the five patients that had up to three family sessions.

**Table 4:** Treatment effects: frequency, distress, disruption, and confidence regarding presenting problem in family consultation (FC)

Measure	Clients Pre-FC Mean (SD)	Clients Post-FC Mean (SD)	n	t	*p*-value	95th CI	ES (d)	Carers Pre-FC Mean (SD)	Carers Post-FC Mean (SD)	n	t	*p*-value	95th CI	ES (d)
Frequency	7.39 (2.92)	6.30 (2.54)	20	1.504	0.147	− .43 to 2.61	0.34	7.32 (2.01)	6.27 (1.90)	17	4.518	0.003*	.56 to 1.56	1.09
Distress	7.02 (1.80)	6.19 (2.11)	21	1.427	0.235	− .38 to 2.05	0.31	7.89 (1.60)	6.31 (1.82)	18	4.123	0.002*	.77 to 2.39	0.97
Disruption	7.34 (2.34)	5.26 (2.83)	19	3.254	0.003*	.74 to 3.42	0.75	6.94 (2.13)	5.97 (1.99)	17	2.697	0.013*	.21 to 1.73	0.65
Confidence	4.74 (2.50)	6.05 (1.72)	21	− 3.005	0.010*	− 2.22 to − .40	− 0.66	6.31 (2.30)	6.56 (1.82)	16	− 0.409	0.481	− 1.55 to 1.05	− 0.11

**p ≤ 0.05; *SD* standard deviation, *ES* effect size, *n* sample size, which varied due to missing data, *CI* confidence intervals

### Prior to family involvement

Identified problems that families wanted to address in the consultation session occurred at a similar frequency across both patient and carer groups, with a mean of 7.39 (SD 2.92) and 7.32 (SD 2.01) respectively (as measured on a 10-point scale where 1 indicates ‘never’ and 10 ‘all the time’). Prior to family consultation, self-reported levels of distress about the problems (‘How upset/worried are you about these issues at the present time?’) was also similar with carers reporting corresponding levels of worry (mean 7.89; SD 1.60) to patients (mean 7.02; SD 1.80). The level of disruption caused by the problems (1 indicating ‘not at all’ and 10 ‘dominating my life completely’) was also similar for patients (mean 7.34; SD 2.34) and for their carers (6.94; SD 2.13). Pre-session self-reported confidence to deal with the issues (1 indicating ‘not at all confident’ and 10 ‘extremely confident’) was higher in carers (mean rank of 6.31/10) than patients (4.74/10).

### After family involvement

Reported frequency of problems was lower for both groups of participants following family consultation. Statistically significant differences between the pre-consultation (mean 7.32; SD 2.01) to post-consultation (mean 6.27; SD 1.90) scores were found for carers [t(16) = 4.52, *p* = 0.003], but did not reach significance in the patient group. Distress in carers also reduced significantly from before (mean 7.89; SD 1.60) to after (M = 6.3, SD = 1.8) the family session; t(17) = 4.12, *p* = 0.002. In addition, the level of life interference or disruption caused by the problems was less post-intervention for both patients [pre-session mean 7.34; SD 2.34; post-session mean 5.26; SD 2.83; t(18) = 3.25, *p* = 0.003] and carers [pre-session mean 6.94; SD 2.13; post-session mean 5.97; SD 1.99; t(16) = 2.69, *p* = 0.013]. Whilst the change in confidence score from pre to post-consultation for the carer group did not reach significance, patients reported a significant increase in confidence to deal with the identified issues following the family session (pre mean 4.74; SD 2.50; post mean 6.05; SD 1.72; t(20) =  − 3.01, *p* = 0.010).

For patients, medium-sized effects of the intervention on measures of disruption caused by (i) the interpersonal problems and (ii) confidence to deal with the issues discussed in the family session, were found (0.75 and − 0.66 respectively) [69]. Moderate to large-sized effects of the family intervention were found for measures of frequency of problems and distress and disruption resulting from the family issues (1.09, 0.65, and 0.97) for carers.

Analysis of individual trajectories revealed a more complex picture regarding the impact of family consultation during treatment than when reporting results as mean change in participant groups. For instance, even though there was a non-significant change in the average rating of patients’ self-reported distress as a result of the identified issues pre-and post- meeting, subgroup differences revealed 11 of the 21 of patients reported a drop in their level of worry, six participants reported a slight increase, and four showed no change over time. This individual variability in the data suggests that the impact of a family meeting on self-reported distress varies, as would be expected in a heterogeneous adult clinical population. For carers, individual trajectory analysis reveals that whilst most carers reported their confidence increased (9/16), three stayed at the same level, and four reported a drop in confidence. Open text answers revealed that these families identified different priority problems between patients and their carers and this drop in confidence could be a result of the carer’s exposure to the patient’s actual issues for the first time.

A majority of both patients (16/20) and carers (15/18) confirmed that the issues were either discussed adequately or fully addressed in via the brief family intervention. For example, “We did talk about this a lot, so it was addressed which was good because I find this hard to discuss with him” (Patient 12). And “All points were addressed quite well in the limited time. The last point however, I feel was address the best – we now have set guidelines for this, and I found it extremely helpful” (Mother, Carer 14). The remainder gave equivocal responses. For instance, in reply to the question ‘To what extent do you feel these questions/issues were addressed?’, one patient stated “We talked about things, but it is still hard at home. Not everything is fixed but they are a bit more aware” (Patient 29). Several respondents also noted the longer-term effect of addressing their presenting problems. See Table 5.

**Table 5:** Acceptability of brief family consultation for addressing participant presenting problems

Patient responses	Carer responses
Discussed	
They were well covered. I am very pleased with the issues we discussed.	Pretty good. We did talk about her experiences and how I can help better.
We talked about that. It was hard though.	Well discussed at meeting.
Was discussed. Given things to try but still difficult.	It was good that we definitely talked about this and made a plan.
*Addressed*	
I thought these questions/issues were addressed very well. We both felt supported and were given the chance to voice our opinions. I felt listened to and respected.	These questions were addressed well. We went through them quite methodologically and had ample opportunity to address other questions that arose
They were addressed and sorted out.	All points were addressed quite well in the limited time. The last point however, I feel was address the best—we now have set guidelines for this and I found it extremely helpful
They were addressed reasonably well but there was a larger focus on my family's understanding of my ED rather than my experience of the ED.	During the session topics were raised and discussed, but not answered. But during the break {partner} and I successfully continued the conversation around maintaining weight.
I felt the session thoroughly addressed my concerns. These were addressed effectively and I was pleased that all topics were able to be covered.	We've discussed but haven't been able to implement as {daughter} very unhappy all the time.
We did talk about this a lot so it was addressed which was good because I find this hard to discuss with him.	They were addressed but she still has the eating disorder.
They were addressed well.	The issue regarding communication was addressed most fully—particularly in relation to the program. The suggestion that we schedule a weekly meeting was taken on-board by the three of us.
Covered well/fully addressed.	
*Effect of addressing presenting problems/outcomes*	
Plan in place to have bi-weekly meetings together. {Partner} understands his responsibilities. I agree with what needs to be done each week in terms of planning. Able to be assertive of my needs and open with {partner}.	Have had family meetings Sunday night. Are talking more openly about things.
I felt my parents were educated a bit more.	It was particularly useful to set up a regular (time-limited) meeting with {daughter} to facilitate discussion and to receive feedback about her week on the program. Doing this in the family session made it more likely to happen! I think there will be a carry-over of this more generally.
I feel that I put what I needed on the table and now we are more. focused on moving forward.	Some good strategies have been suggested (and been used to good effect) but there's quite a lot of stress and it will take a long time to get back to a more relaxed state.
We focused a lot on creating and opportunity to have a deliberate conversation each week about the program and my ED—I found this beneficial. We did not get to cover ways of coping outside the Day program.	This was done, that is how to communicate better, and we now have a way to talk about things, a regular time, and we have started doing that.
Break through moment, very necessary in continuing to answer these questions. Has helped in breaking the ice- easier for both of us to reach out now, even for trivial things.	We talked about what my role is, i.e., to offer moral support, solidarity, more prompting/reminding not policing and breakfast strategies.
I feel that we addressed things very well. As partially a result from the family consult, I feel incredible committed to my recover and meeting the weight target at QuEDS. I was especially rational the day following the family consult.	It was a really good session. To have both the therapists there and to get to hear about how {daughter’s} treatment is going was great. I’m so happy she’s finally getting some help.
Addressed quite well and helped open a gate for more communication outside.	I think {daughter} agreed, and I am hoping, that she will let me know when and if she needs my help. I don't see her every day, but we catch up almost daily.
We talked about things but it is still hard at home. Not everything is fixed but they are a bit more aware.	

In addition, seven patients and nine carers described outcomes that included enhanced communication and flow on motivational and relational effects from the intervention. For example, one patient wrote that, “{Partner} understands his responsibilities. I agree with what needs to be done each week in terms of planning. Able to be assertive of my needs and open with {partner}” (Patient 8), and a carer noted, “It was particularly useful to set up a regular (time-limited) meeting with {daughter} to facilitate discussion and to receive feedback about her week on the program. Doing this in the family session made it more likely to happen! I think there will be a carry-over of this more generally” (Father, Carer 14).

### Other changes following the intervention

Other changes following the intervention included communication (13 patient and 12 carer responses), helpful actions (6 patient and 4 carer responses), general support related to the ED (4 patient and 4 carer responses), emotional assistance (6 patient and 1 carer response) and understanding about the ED (3 patient and 1 carer response). For example, a patient (23) stated she now received “More frequent messages from Dad providing encouragement, and first call from dad, just to chat. Able to reach out when things aren’t good and knowing he’ll listen”. Another said that “Not talking about triggering topics while eating is extremely helpful. More meal planning so I can prepare for the meal ahead. Post treatment support from family and friends have been great” (Patient 14). A carer stated that.

{Daughter’s} attitude to wanting to get better appears to be changing. Each week we have met over coffee for a specified length of time and discussed the program. She has been accommodating in terms of sharing activities that have taken place and explaining certain expectations, as yet there is little discussion about how she is feeling or coping. Generally, I think that sees this program as a collaboration between herself, the QUEDS team and my husband and I. Almost all previous therapies have tended to pay lip-service to our involvement and {daughter} has often reinforced that that is how she would prefer it to be.” (Mother, Carer 13)

Concerns that participants felt had not changed, fell into similar categories, that is, communication (5 patient and 3 carer responses), helpful actions (1 patient and 1 carer responses), general support related to the ED (2 patient and 4 carer responses), and emotional assistance (5 patient and 5 carer response) indicating some difficulties may take longer to shift. As one carer put it, “We’re still having fights. I feel bad about it” (Partner 20), and a patient stated, “I still find it hard to tell all the things to my mum as I don’t want to worry her” (Patient 5).

### Participant needs following the intervention

When asked to list any further needs or support required, a majority of participants did not identify any problems indicating sufficiency of the single session to meet their needs in relation to the priority problem. Four participants asked for resources such as more reading, psychoeducation materials, or information regarding future treatment. Two patients and two carers asked about the possibility of having further family sessions.

### Other feedback about the intervention

Finally, ‘other’ comments regarding the family consultation were provided by 12 participants. Each of these was either a note of thanks or a positive remark regarding the session. For example, one patient (8) stated, “Great session together, well-structured and we both felt heard” and another said, “The session helped me feel supported and heard by {partner}. It made me less fearful about any damage I caused because of my eating disorder” (Patient 17). One mother wrote “I appreciate the time for the family session and for the support {patient} got from the program” (Carer 9).

## Discussion

Research into the use of family interventions with adults with ED is sparse. Results of the current study confirm that the concept of brief, low intensity family work is of interest and beneficial to adult patients with EDs and their carers as well as being feasible to implement in a real-world clinical setting. Participant self-report before and after changes supported the assumption that family intervention, in many cases as brief as one session, may be enough to assist families in the areas they identify as important. Outcomes for those who requested further family sessions (up to three during treatment) were also similarly positive.

The method for brief family consultation (applied as per the protocol for other mental illnesses, [45], was able to be successfully operationalised in a standard adult EDs outpatient treatment setting, with little customisation required. No adverse events were recorded during the trial. Implementation fitted the prevailing requirements and resources of a busy outpatient service offering standard care and was supportive of the existing therapeutic relationships, treatment approaches, and clinical competencies found within the standard multidisciplinary EDs team.

The high uptake of the brief family intervention indicates that even a single opportunity to work on relational problems was welcomed by adult ED patients and their carers and appeared to offer participants a way to lessen the impact of current concerns they identified, possibly by reducing the frequency with which they occur. Patients also reported confidence to deal with problems significantly improved following the family meeting, carers reported feelings of worry were significantly less, and the extent to which problems were interfering in life was rated as significantly lower for both patients and carers.

The active ingredients of the brief intervention that contributed to these reported benefits were unable to be determined by the current study design, but it may be that by allowing participants to approach a previously avoided topic in a planned way, via a structured setting, the family session galvanized participants’ own strengths. The demonstrated utility of the approach‐avoidance goal distinction, and its applicability to motivation and mastery experiences may be particularly relevant in EDs as self-efficacy problems affect both patients and carers [70–72]. Allowing patients to choose to involve carers, control which support include, and how they are engaged, could create an opportunity for patients to exercise self-determination. This, in addition to the structure and the active, collaborative participation central to brief approaches, may provide participants with a mastery experience around previously avoided relational difficulties. Self-determination theory suggest this happens via a sense of volition and effectiveness as well as connecting with supportive others [73]. Maximizing autonomy by facilitating patient choice and providing for patient values and preferences where possible, has also been shown to be important treatment considerations in EDs [2, 74].

For carers, the amount of external support available is known to improve coping and reduce strain [75]. Increasing this by the offer of treatment involvement, even with only a minimal level of service support, could have affected the interactions between patient and carer producing a more positive cycle [76]. ‘Service support’ has been hypothesized to be a necessary part of appraising the impact and adapting to the experience of caring for an individual with an ED [77].

Conceptually, a central tenant of the framework used in the current study is the collaborative relational approach it offers and the highly structured, self-determined, and solution-focused processes it incorporates ([45], The Bouverie Centre [78]). This combination, known to be effective in other areas of mental illness [37, 49, 51, 52, 79], may also be suitable for use with adult EDs, where little attention has been paid to the routine engagement and inclusion of families.

The lack of research evidence to inform clinical guidelines regarding how clinicians might best work with the families of adults with EDs, highlights a theory–practice gap as well as a pragmatic problem for service providers in the field. Without a therapeutic frame establishing the ‘rules of engagement’ with the carers of adults, treatment services may be at risk of operating without the benefit of stated boundaries and the safety this provides to both clinicians and clients. The less than optimal experiences of the sector reported by families could conceivably be the result of this lack of structure or planning when working with carers [77, 80].

Caution should be applied to extrapolating too far from the results presented however given the small, heterogeneous sample used and the fact that the study was not experimentally controlled. In addition, it could be that the changes reported by participants were the result of treatment in general rather than family inclusion or that the families that chose to have consultations did so when there were experiencing greater difficulties and the changes reported are regression toward the mean. The measures used also relied on self-report and were inherently attitudinal so social desirability bias may have been operating, although a broad range of participant responses to the open text questions was received that included both positive and negative views regarding the elements of the intervention.

Despite these limitations, the results suggest that brief, patient-directed consultation with adult families affected by ED utilising a standardised structure does appear feasible and could provide a minimal level of acceptable service responses to working with the families of adults. This method could also provide well an appropriate contrast in future studies with more robust comparative designs [81, 82]. For example, the acceptability, perceived relevance, and utility of brief interventions within a range of family support options for adult EDs utilising differing intensity could be evaluated [83, 84]. A stepped care model for working with families has been developed in the general adult mental health services sector and could also be usefully applied to EDs [30].

## Conclusion

Current clinical guidelines recommend that family members or other supports be routinely engaged by clinicians working with adults affected by EDs in order to both support carers in their role and provide assistance regarding current relational issues that may affect treatment. If supported by future empirical findings, the structured, patient-directed approach investigated in the current study may offer a feasible and effective means for standardising the involvement of the natural support system in the outpatient ED treatment of adults, bringing the field in line with advances in in other areas of equivalent serious mental illness.

## Data Availability

The dataset collected and analysed in the current study are not publicly available due to ethical restrictions to maintain the participants’ anonymity. The corresponding author can be contacted on reasonable requests regarding the dataset.

## Abbreviations

AN: Anorexia nervosa
BN: Bulimia nervosa
DSM-5: Diagnostic and statistical manual of mental disorders, 5th edition
ED: Eating disorder
EDs: Eating disorders
EDE-Q: Eating disorder examination questionnaire
EDNOS: Eating disorder not otherwise specified
FIT: Family inclusive treatment
OSFED: Other specified feeding and eating disorder
QuEDS: Queensland eating disorder service
SSFC: Single session family consultation
TAU: Treatment as usual
